# The Ring Rotation Illusion: Properties and Links of a Novel Illusion of Motion

**DOI:** 10.1177/20416695211020019

**Published:** 2021-06-07

**Authors:** Uwe Mattler, Maximilian Stein, Robert Fendrich

**Affiliations:** Department of Experimental Psychology, University of Goettingen, Goettingen, Germany; Dartmouth College, Hanover, New Hampshire, United States

**Keywords:** Ring Rotation Illusion, Motion Bridging Effect, unconscious perception, apparent motion, heuristics

## Abstract

We report a novel visual illusion we call the *Ring Rotation Illusion* (RRI). When a ring of stationary points replaces a circular outline, the ring of points appears to rotate to a halt, although no actual motion has been displayed. Three experiments evaluate the clarity of the illusory rotation. Clarity decreased as the diameter of the circle and ring increased and increased as the number of points forming the ring increased. The optimal interstimulus interval (ISI) between the circle and ring was 90 ms when stimulus presentations lasted 100 ms but 0 ms with 500 ms presentations. We compare the RRI to the Motion Bridging Effect (MBE), a similar illusion in which a stationary ring of points replaces an initial ring of points that spins so rapidly it looks like a stationary outline. A rotation of the stationary ring is seen that usually matches the direction of the initial ring’s invisible spin. Participants reported a slightly more frequent and clearer motion percept with the MBE than RRI. ISI manipulations had similar effects on the two illusions, but the effects of number of points and ring diameter were largely restricted to the RRI. We suggest that both the RRI and MBE motion percepts are produced by a visual heuristic that holds that the transition from an outline circle to a ring of points is plausibly explained by a rapid spin decelerating to a halt, but in the case of the MBE, an additional direction-sensitive mechanism contributes to this percept.

In 2010, Mattler and Fendrich reported an illusion of motion that implies the human visual system can process temporal frequencies substantially more rapid than those that can mediate the perception of flicker (∼60 Hz; e.g., Landis, 1954) and motion (∼30 Hz; Burr & Ross, 1982). A ring of 16 luminous points was rotated so rapidly observers perceived a continuous circular outline and performed at chance when asked to report the rotation direction. However, when this rapidly rotating ring was replaced by a static ring of 16 points, the static ring appeared to briefly spin, predominantly (up to 82% of the time with a 1500°/s angular rotation velocity) in the same direction as the rotating ring.

Mattler and Fendrich labeled the initial veridically rotating but apparently static ring as the *inducing ring* and the veridically static and apparently rotating subsequent ring as the *test ring*. Because the illusory test ring rotation was linked to the inducing ring rotation, they termed this illusion the *Motion Bridging Effect* (MBE). The congruence between the direction of the inducing and illusory test ring rotations indicates that, although it is not consciously detectible, the direction of the inducing ring spin must somehow be encoded. This is the case despite the fact that at every inducing ring location where a point is displayed the advancing points of the inducing ring may be refreshed at rates of up to 125 Hz (Stein et al., 2019).

Mattler and Fendrich presumed that the percept of motion in the test ring was per se conveyed by the inducing ring’s spin. However, a continued investigation of the MBE’s functional dependencies (Stein et al., 2019) raised the possibility that the motion percept and motion direction information were in fact dissociable signals. This led to the speculation that while the direction information must be derived from the inducing ring spin, the actual motion percept might represent an instance of apparent motion produced by the transition from an apparently continuous to a point defined ring outline.

An implication of this view is that the motion of the inducing ring should not be necessary for the illusory motion percept to occur. That is, if a truly continuous stationary outline circle replaces the spinning inducing ring, a motion percept should still be seen in the test ring. In the experiments we report here, this prediction is confirmed. We term the illusion of motion generated by the veridically continuous stationary inducer as the *Ring Rotation Illusion* (RRI). For consistency, when discussing the RRI, we adopt the nomenclature used to describe the MBE and refer to the *inducing ring* and *test ring*. Note that our use of the “inducing ring” label indicates only that the outline circle is required to produce the illusory motion percept in the test ring and is not intended to have implications regarding the character or mechanism of the illusion. To designate the crucial difference between the two types of display—the presentation of a rapidly rotating inducing ring in the case of the MBE and a continuous stationary outline in the case of the RRI—we refer to the *rotating* and *stationary* inducers.

## Overview

We report three experiments. In the first, we investigate some basic stimulus dependencies of the RRI, specifically, how it is affected by manipulations of the diameter of the rings, the number of points used to form the test ring, and ISI between the ring presentations. A second experiment compares the clarity of the illusory motion percept generated by the RRI’s stationary inducer and the MBE’s rotating inducer to evaluate the premise that both percepts are mediated in the same manner. A third experiment extends this evaluation by comparing the effects of the stimulus manipulations investigated in the first experiment with the two display types.

## General Methods and Apparatus

In all three of the reported experiments, stimuli were displayed on an analog HAMEG HM 400 cathode-ray oscilloscope controlled by a PC with a 12-bit digital-to-analog converter. The 8 × 10 cm oscilloscope screen was customized with a very short persistence P15 phosphor (50 µs luminance decay time to 0.1%) so that stimulus offsets on the CRT screen were nearly instantaneous. Participants sat in a dark room with their head positions stabilized by a chin and forehead rest 57 cm from the oscilloscope screen.

## Experiment 1

Following initial observations of the RRI in which we confirmed that an illusory rotation was seen when a ring of points replaced a stationary outline circle, we conducted Experiment 1 to evaluate some characteristics of this illusion. Specifically, we measured participants’ ratings of the clarity of the perceived test ring rotation as a function of the number of points in the test ring, the diameter of the rings, the duration of the ring presentations, and the ISI between the inducer and test ring.

### Method

#### Participants

Twelve students of the University of Göttingen participated in the Experiment with an average age of 26.4 years (8 females, 4 males). Nine of them reported they were right-handed, and three reported they were left-handed. All participants had normal or corrected-to-normal vision as determined by the Landolt ring chart and received €7 per hour or student credits. Each participant completed two 1-hour sessions, which were run on separate days.

#### Stimuli

**Figure 1.**
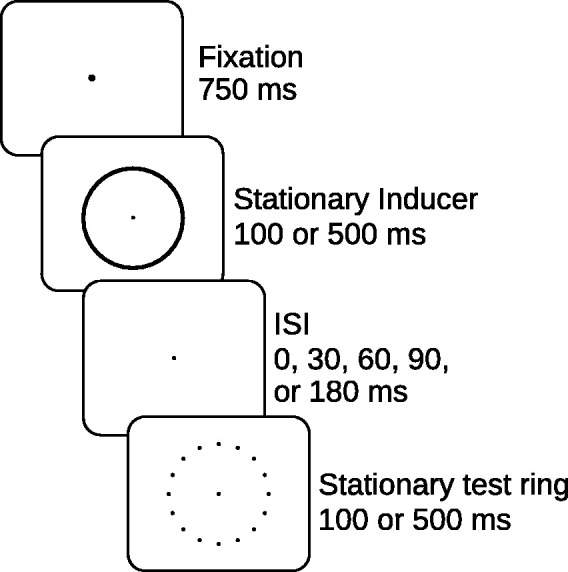
Sequence of events in Experiment 1. Note, on the oscilloscope screen, stimuli were bright on a dark background. ISI = interstimulus interval.

On each trial, a stationary outline circle (the stationary inducer) was followed by a stationary ring of points (the test ring). The sequence of events is shown in Figure 1. There were 1,440 potential display positions around the circumference of the stationary inducer. The stationary outline circle was displayed using a rapid refresh rate algorithm that prevented incidental local motion signals from occurring. Specifically, 160 points were displayed at a randomly selected subset of the 1,440 outline circle locations every millisecond. The random selection of point positions occurred without replacement so that each of the entire 1,440 display positions was shown before any point was displayed again. This resulted in a point being displayed at every circumference location with an average temporal frequency of 111 Hz. Therefore, every outline circle position was refreshed at a rate substantially higher than the human critical flicker fusion frequency, eliminating detectable local luminance fluctuations. In addition, the central fixation point was displayed 10 times per millisecond.

The standard diameter of the rings (when diameter was not serving as an experimental variable) was 6° of visual angle. When the number of points in the test ring was not serving as an experimental variable, the test ring was constructed of 16 equally spaced luminous points. When the ISI was not serving as an experimental variable, its standard duration was 60 ms. All points were slightly out of focus on the oscilloscope screen because we previously found this strengthened the illusory motion percept being studied. The brightness of the stationary outline circle was 0.099 cd/m^2^, and the point brightness of the test ring was 1.20 cd/m^2^ on a dark background. The methods employed to obtain these brightness measurements are described in Stein et al. (2019).

#### Procedure and Design

In the first session, the duration of each of the ring presentations was 500 ms and the ISI was 60 ms. The session began with an initial practice block that was followed by eight 48 trial experimental blocks. In the first four blocks, we varied the number of points in the test ring within each block with 8, 12, 16, or 20 equally spaced points displayed. In the next four experimental blocks, we varied the diameter of the two rings between the stimulus blocks (with the two rings always having the same diameter on any trial), with diameters of 2°, 4°, 6°, and 8° of visual angle following a Latin Square Design. During the second session, a practice block was followed by 10 experimental blocks of 50 trials. Within each block, we quasi-randomly varied the ISI between the two stimuli with ISIs of 0, 30, 60, 90, and 180 ms. The duration of the ring presentations was 500 ms in the first half of these blocks and 100 ms in the second half.

Participants were instructed to report the clarity (German “Deutlichkeit”) of the rotation that they perceived after the presentation of the two-stimulus displays on a modified computer keyboard with seven keys in a horizontal row that had the numbers 3, 2, 1, 0, 1, 2, 3 printed on them. The keys on the left indicated a counterclockwise rotation and those on the right a clockwise rotation, with “3” indicating a maximal, “2” a middle, and “1” minimal clarity. The middle key was assigned the value zero, and participants were instructed to use this key when they could not specify a rotation direction. The space bar below this row of keys was used to start the next block of trials. Ratings were collapsed across clockwise and counterclockwise directions to determine the average clarity rating for each participant in each experimental condition.

#### Statistical Analyses

Forty-eight (50) trials were evaluated in each condition of the first (second) session. The average clarity ratings of each participant in each condition were analyzed with three repeated measures analyses of variance (ANOVAs): one evaluating the effect of *Number of Points*, one evaluating *Diameter*, and the third evaluating the independent variables *ISI* and *Stimulus Duration*. Degrees of freedom were corrected using Greenhouse–Geisser estimates of sphericity, but for the sake of readability, the uncorrected degrees of freedom are reported.

### Results

**Figure 2.**
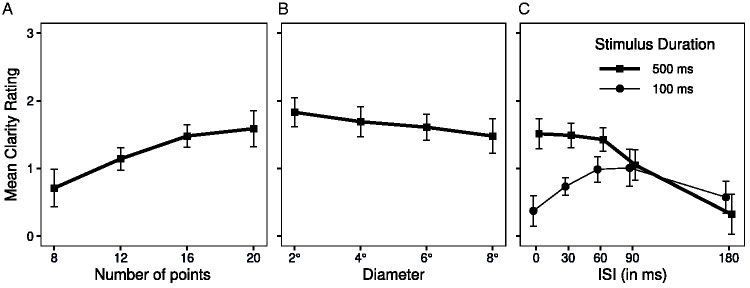
Mean clarity ratings of the Ring Rotation Illusion in Experiment 1 as a function of (A) the number of points in the test ring, (B) the diameter of the rings, and (C) the interstimulus interval (ISI) between the inducing and test ring presentations for ring presentation with durations of 100 and 500 ms. In both A and B, the duration of the two rings was 500 ms. The error bars show 95% confidence intervals for the within-subject difference in means (Cousineau, 2005; Morey, 2008).

The results of Experiment 1 are presented in Figure 2. Overall, a clockwise rotation was reported on 50.83% of the trials and counterclockwise on 18.85%. On the remaining 30.32% of the trials, participants were unable to specify the motion direction. This could have occurred because they did not perceive any motion or because they perceived a spatial or temporal mix of motion directions, so that neither a clockwise nor counterclockwise response was exclusively correct. In Experiment 2, where participants could distinguish these alternatives, “mixed motion” percepts were, in fact, reported a little more often than “no-motion” percepts.

#### Number of Points

The main effect of *Number of Points* was significant, *F*(3, 33) = 14.94, *p* = .002, η_p_^2^ = .576, with the clarity of the rotation increasing as number of points increased (Figure 2A).

#### Diameter

Mean clarity ratings trended downward when the ring diameter increased (Figure 2B), but this trend failed to reach statistical significance, *F*(3, 33) = 2.13, *p* = .121, η_p_^2^ = .162.

#### ISI and Duration

The main effects of *ISI* and *Stimuli Duration* were both significant, *F*(4, 44) = 20.55, *p* < .001, η_p_^2^ = .651, and *F*(1, 11) = 18.64, *p* = .001, η_p_^2^ = .629, respectively, and the interaction of these factors was significant, *F*(4, 44) = 19.30, *p* < .001, η_p_^2^ = .637 (Figure 2C). Overall, clarity was greater with the 500 ms ring durations than 100 ms ring durations, but there was a pronounced interaction of ISI with duration. With 100 ms stimulus duration, clarity was at its *minimum* when the ISI was 0, increased to a maximum at 60–90 ms, and then declined. With 500 ms stimulus duration, the clarity was at its *maximum* with 0 ms ISI, stayed relatively stable to 60 ms, and then declined.

### Summary

In Experiment 1, we found the clarity of the RRI increased as the number of points in the test ring increased and was modulated by the ISI between the inducing and test rings in a manner that was strongly dependent on the duration of the ring presentations. We also found motion clarity was better with smaller diameter rings, but this effect was statistically nonsignificant. We note, however, that in Experiment 3 (reported later), in which a shorter stimulus duration was employed, a similar effect of diameter did reach significance.

The source of these effects remains to be determined, but we note that both increasing the number of points and decreasing the ring diameter have the effect of reducing the separation between the test ring points, raising the possibility that this could be a mediating factor in both cases (see Stein et al., 2019). We also note a link between our data and a finding in the traditional apparent motion literature. Kahneman (1967) reported an effect of stimulus durations on apparent motion that parallels our ISI findings. An optimal apparent motion was found with an ISIs of 75–100 ms when stimulus durations were 25–125 ms but an ISI of 0 ms when stimulus durations were 800 ms. We return to the possibility that the RRI is a form of apparent motion in the general discussion.

## Experiment 2

To the extent that the rapidly rotating inducing ring of the MBE display and stationary inducer display are visually indistinguishable, it might be argued that the RRI and MBE are simply instances of the same illusion. We believe, however, that there are strong grounds for distinguishing between the MBE and RRI. A crucial aspect of the MBE is the transfer of invisible direction information from the inducing ring to test ring, and this attribute has no RRI counterpart because with the stationary inducer there is no inducing ring motion. The core measure previously used to quantify the MBE – the degree of congruence between the actual direction of the rapidly rotating inducing ring and the direction that is perceived in the test ring – therefore cannot be applied to the RRI. If the MBE is deemed to have two components, one that generates the illusory test ring motion and one that biases the direction of that motion, the motion generating component may well be common to the two illusions, but the direction biasing component will be unique to the MBE. In this case, it would be reasonable to expect the illusory rotation generated by these displays to be equivalent if one disregards motion direction. This is not, however, a necessary equivalence. The rotation of the inducing ring in the MBE display might not just bias rotation direction but also act to augment the motion percept that the MBE and RRI share. In Experiment 2, we evaluated this possibility by empirically determining whether the rotating MBE and stationary RRI inducing rings were genuinely indistinguishable and then examining whether the illusory motion percepts generated by the two types of inducer had the same degree of clarity. Data we report suggest some augmentation of the illusory motion by the inducing ring spin does in fact occur.

### Method

#### Participants

Twelve new University of Göttingen students (10 females, 2 males) with an average age of 24.1 years participated in the Experiment. Nine of them reported they were right-handed, and three reported they were left-handed. All had normal or corrected-to-normal vision as determined by the Landolt ring chart and received €7 per hour or student credits. Each participant completed three 1-hour sessions, which were run on separate days.

#### Stimuli

**Figure 3.**
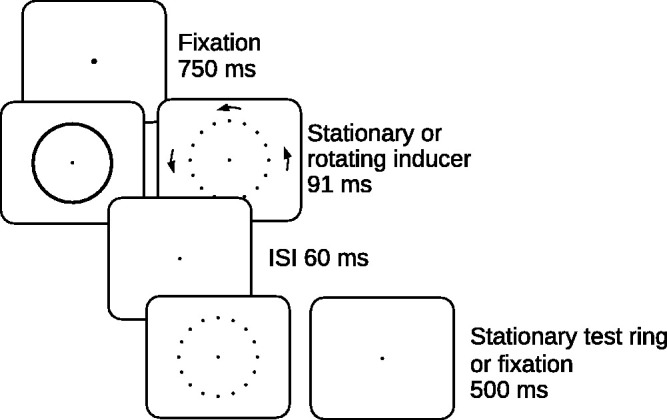
The stimulus sequence in Experiment 2. On a given trial, the inducing ring rotated either counterclockwise (as shown in the figure) or clockwise. The points on the oscilloscope were bright on a dark background. The start and stop positions of the inducer points always matched the test ring point positions. ISI = interstimulus interval.

The stimulus display sequence is shown in Figure 3. In all the experimental sessions, either the *stationary inducer* or *rotating inducer* was initially presented for 91 ms. Inducer Type was varied randomly between trials. The diameter of the rings was 7.5° of visual angle. The stationary inducer was created in the manner described in Experiment 1. The rotating inducer was rotated by concurrently advancing 16 equally spaced points every millisecond by a specified number of steps around the 1,440 possible point locations on the ring circumference. This updated the display with an effective frame rate of 1000 Hz. The rotation was clockwise or counterclockwise at angular velocities of 250 or 1500°/s. These rotation rates were respectively achieved by advancing the points by 1 or 6 steps each frame and resulted in points being refreshed at each utilized display position on the inducing ring circumference at temporal frequencies of 11.11 and 66.67 Hz. At the 1500°/s rotation speed, the inducing ring appeared (like the stationary ring) to be a continuous outline circle. The time-averaged brightness of inducing ring points was 0.017 and 0.099 cd/m^2^ at the 250 and 1500°/s velocities, respectively. Note that the location and timing of the point presentations in the stationary and rotating rings was identical when the velocity of the rotating ring was 1500°/s, with the only difference being that the sequence of point positions in the stationary ring was scrambled. In Sessions 2 and 3, after a 60-ms ISI, the stationary 16-point test ring followed the inducer for 500 ms. The test ring point brightness was 1.50 cd/m^2^ on a dark background.

#### Procedure and Design

In the first session, we examined participants’ ability to distinguish the rotating inducer from the stationary inducer. Participants indicated whether the stimulus was stationary or rotating, irrespective of the rotation direction, by pressing one of two keys. In the first four blocks, the inducer rotated with a low velocity of 250°/s; in the following four blocks, the velocity was 1500°/s. The first block of each group of four blocks was considered practice and excluded from analysis. Across the remaining six experimental blocks of 48 trials each, we gathered 144 trials for each velocity. On half of the trials in which the rotating inducer was presented it turned clockwise, and on the other half it turned counterclockwise.

In Sessions 2 and 3, the stationary ring of points (*test ring*) followed the inducer (see Figure 3). The inducing ring was presented for 91 ms, and the test ring was presented for 500 ms. The stationary and the rotating inducer were each shown on half the trials. On half of the trials in which the rotating inducer was presented it turned clockwise, and on the other half it turned counterclockwise. The velocity of the rotating inducer was always 1500°/s. In one of the two sessions, participants were asked to rate the clarity of rotation. In the other session, to obtain a second supporting measure of the strength of the perceived rotation, participants were asked to judge the distance the points moved during the perceived rotation. The order of the two tasks was balanced across participants.

Ratings of motion clarity and distance were given after the presentation of the second stimulus. Five response keys were arranged in a horizontal line on a modified computer keyboard, with the leftmost key indicating a very clear (very far) rotation in counterclockwise direction, the rightmost key a very clear (very far) rotation in clockwise direction, and the middle key a “rotation in both directions.” The remaining two keys were assigned to less clear (less far) counterclockwise and clockwise rotations. Below this line of keys, there was the space bar that allowed subjects to report “no-motion” percepts. Following a practice block, in each of the sessions, eight blocks of 48 trials each were run, providing 192 trials of data for both the stationary and the rotating inducer.

#### Statistical Analyses

To evaluate participants’ ability to distinguish between the stationary and rotating inducers, we performed a signal detection analysis (Macmillan & Creelman, 2005). “Rotating” responses to rotating inducers were considered hits, and “rotating” responses to stationary inducers were considered false alarms. Hit and false alarm rates were determined for each subject in each condition, and the log-linear correction rule of Hautus (1995) was applied. To compare the strength of the RRI and MBE illusory motion percepts, we respectively assigned rating values of 0, 1, and 2 to participants’ “no” or “bidirectional motion”, less clear (less far), and very clear (very far) responses. Mean rating values for clockwise and counterclockwise rotations in each of the inducer conditions were calculated and then collapsed across direction. *t* tests were used to evaluate condition-dependent differences in *d′* and the mean clarity and distance ratings. We also contrasted the frequency of the reports of “no motion” or “motion in both directions” in the conditions when the stationary and rotating inducer had been presented using two-tailed Wilcoxon signed-rank tests to meet the nonnormal distribution of these variables. Finally, to determine whether there was an MBE when the rotating inducer was displayed, we determined the frequency of direction reports that were congruent with the actual spin direction of that inducer.

### Results

#### Discrimination of the Inducer Type

**Figure 4.**
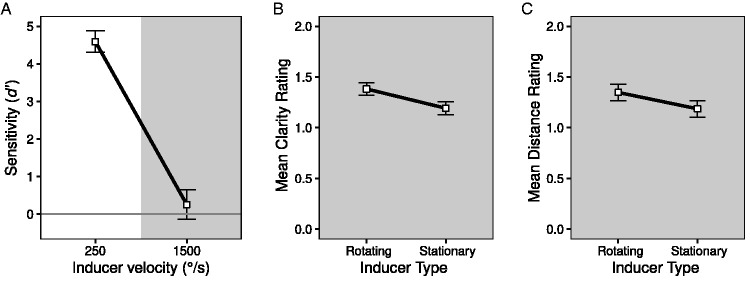
Strength of the Ring Rotation Illusion in Experiment 2. (A) Participants sensitivity to the difference between the rotating and stationary inducer when the rotation velocity was low or high. (B) Mean rating of the clarity of the rotation for stationary inducers and rotating inducers with a velocity of 1500°/s. (C) Mean rating of the distance covered by the rotation for stationary inducers and rotating inducers with a velocity of 1500°/s. The error bars show 95% confidence intervals for single means in A, and for the within-subject difference in the means in B and C (Cousineau, 2005; Morey, 2008).

Participants were perfect at distinguishing the stationary from rotating inducer when the rotation velocity was 250°/s, *t*(11) = 35.45, *p* < .001, but performed at chance when the velocity was 1500°/s, *t*(11) = 1.42, *p* = .09, with respective mean *d'*s of 4.59 and 0.25 (see Figure 4A).^1^

#### The Incidence of Motion Percepts in the Clarity Task

Clockwise and counterclockwise rotations were, respectively, reported on 49.39% and 30.73% of the RRI trials and 50.56% and 38.54% of the MBE trials in which participants reported the motion clarity. Of the remaining 19.88% of the RRI trials, 11.16% were reports of a “rotation in both directions” and 8.72% were “no-motion” reports. Of the remaining 10.9% of the MBE trials, 6.38% were reports of a “rotation in both directions” and 4.52% were “no-motion” reports. The increased incidence of bidirectional and no-motion reports in the RRI condition relative to the MBE condition were both statistically significant, *W*(11) = 56, *p* = .014 and *W*(11) = 28, *p* < .02, respectively.

#### Motion Clarity

Mean ratings of the clarity of the RRI are shown in Figure 4B. While ratings were generally similar with the rotating and stationary inducers (1.38 and 1.19, respectively), a slightly greater clarity was found with the rotating inducer which was statistically significant, *t*(11) = 4.73, *p* < .001.

#### Incidence of Motion Percepts in the Distance Task

The distribution of clockwise, counterclockwise, bidirectional, and no-motion reports in the distance judgment trials was similar to that found in the clarity task. Clockwise and counterclockwise rotations were, respectively, reported on 52.73% and 31.86% of the RRI trials and 52.99% and 37.80% of the MBE trials. Of the remaining 15.41% of the RRI trials, 9.94% were reports of a “rotation in both directions” and 5.46% were “no-motion” reports. Of the remaining 9.21% of the MBE trials, 6.56% were reports of a “rotation in both directions” and 2.64% were “no-motion” reports. As was the case with the clarity task, the increased incidence of bidirectional and no-motion percepts in the RRI condition relative to the MBE condition were both statistically significant, *W*(11) = 56.5, *p* = .013 and *W*(11) = 26, *p* < .05, respectively.

#### Distance Ratings

Ratings of the distance the test ring points appeared to move yield a similar outcome to those found for clarity (see Figure 4C). Mean ratings were comparable with the rapidly rotating and stationary inducers (1.35 and 1.19, respectively), but the small advantage with the rotating inducer proved to be statistically reliable, *t*(11) = 3.11, *p* = .010.

#### MBE Congruency Effect

The frequency of congruent responses on trials with the rotating inducer confirmed that it produced the MBE effect. Participants reported perceived rotations in the same direction as the rapid rotation on 67.9 and 69.1% of the trials in the clarity and distance rating sessions, respectively. These correspond to mean *d*′ values of 1.42 and 1.51, respectively.

The mean clarity rating was only a little greater on the MBE trials with congruent direction reports compared to MBE trials with incongruent direction reports (1.58 vs. 1.43), but this difference did prove significant, *t*(11) = 3.47, *p* = .005. The mean distance rating was also slightly greater on trials with congruent versus incongruent perceived directions, but this difference did not reach significance – 1.49 versus 1.43; *t*(11) = 1.09, *p* = .299.

### Summary

Experiment 2 compared the illusory test ring rotation when the inducing stimulus was a stationary outline circle to the illusory test ring rotation when the inducer was a rapidly rotating ring of points (the MBE display). Data from an initial session indicated that participants could not distinguish between the two inducer types when these stimuli were presented without the test ring. In the following two sessions, we asked participants to rate the clarity of the perceived test ring rotation and the distance the points moved during the perceived rotation. While similar ratings were obtained with the two types of inducer, there was a small but significant increase in both the reported clarity and distance estimates when the rapidly rotating inducing ring was shown. The increase in clarity with the MBE display was most pronounced when the direction of the illusory rotation matched the direction of the inducer rotation. In addition, there were significantly fewer cases where no or a directionally ambiguous motion was perceived with the MBE display. While the overall similarity of the motion percepts produced by the stationary and rapidly rotating inducers is commensurate with the supposition that the same process generates both of these percepts, the small but persistent advantage found when the rotating inducer is shown suggests that the rotation of the inducing ring in the MBE display, although not consciously visible, serves to augment the illusory ring rotation. It should also be noted that although clarity ratings were slightly better in the MBE trials with directional congruency than without congruency, this effect was quite small, and congruency failed to have a statistically reliable effect on the distance-traveled ratings. The absence of a more pronounced effect of congruency on the clarity and distance ratings supports the hypothesis that the induction of motion and the processing of the direction signal are separable mechanisms.

## Experiment 3

Experiment 2 provided evidence that generally similar motion percepts are generated by the stationary and rotating inducers. However, clarity and distance ratings were slightly but significantly higher when the inducer was the rapidly rotating ring. A more stringent way of evaluating whether the same process generates the motion percepts with the stationary inducer display and the MBE display is to determine whether those percepts exhibit similar functional dependencies. In Experiment 3, we did this by examining whether the display variables investigated in Experiment 1 – specifically, ring diameter, the number of points in the test ring, and the ISI between the inducing and test ring presentations – had similar effects on motion clarity ratings when the test ring followed the stationary and rotating inducers.

In previous studies, the measure of the MBE has been the congruence between the direction of the invisible inducing ring rotation and illusory test ring rotation (Mattler & Fendrich, 2010; Stein et al., 2019, 2020). This method cannot be used to compare the MBE and RRI because in the case of the stationary inducer display, the inducer does not rotate. In Experiment 3, we therefore used motion clarity ratings such as those employed in Experiments 1 and 2 to compare the two motion illusions. Because our clarity rating scale required that participants indicate the direction as well as clarity of the perceived motion, we were still able to measure direction congruence when the rotating inducer was shown. On these trials, we were therefore able to determine the extent to which congruence and motion clarity covaried.

### Method

#### Participants

Twelve new students from the University of Göttingen (12 females, 0 males) with an average age of 20.9 years participated in Experiment 3. Eleven of them reported they were right-handed, and one reported she was left-handed. All participants had normal or corrected-to-normal vision as determined by the Landolt ring chart and received €7 per hour or student credits. Each participant completed four 1-hour sessions, which were run on separate days.

#### Stimuli

On half of the trials, the stationary outline circle (stationary inducer) was shown, and on the other half, the rotating ring of points (rotating inducer) was shown. The Inducer Type was varied randomly between trials in all sessions orthogonal to the variation of the other independent variables. The stationary inducer was created in the manner described in Experiment 1, and the motion of the rotating inducer was created in the manner described in Experiment 2. Average brightness values were the same as in these earlier experiments. When the rotating inducer was presented, its rotation direction was clockwise on half of the trials and counterclockwise on the other half. The sequence of events corresponded to that used in Experiment 2 (cf. Figure 3) except that the inducer and test ring were presented for 121 ms each. The standard parameters of the stimuli were 16 points, a diameter of 6° of visual angle, an inducing ring velocity of 1500°/s, and an ISI of 60 ms. The brightness of the test ring points was 1.2 cd\m^2^ (as in Experiment 1).

In Session 1, we varied the number of test ring points (8, 12, 16, or 20) randomly between trials. In a given trial, when the rotating inducer was shown it had the same number of points as the test ring. In Session 2, we varied the diameter of the rings (with diameters set to 2°, 4°, 6°, and 8° of visual angle). The diameter was varied blockwise, and the order of conditions was balanced across participants according to a Latin square. In Session 3, we varied the ISI (0, 30, 60, 90, or 180 ms) randomly between trials. In Session 4, the stationary circle and the rotating ring were presented alone, with identical stationary circle presentations but the number of points that formed the rapidly rotating inducer varied randomly between trials (8, 12, 16, or 20 points). All stimulus parameters except for the one being varied were set to the standard values. In all sessions, we considered the initial block of trials as practice, and these trials were not included in the analyses.

#### Tasks

In the first three sessions, participants were asked to report the clarity of the perceived rotation after the presentation of the stimuli. We used the same instructions and modified keyboard as in Experiment 1 in these sessions. In Session 4, participants were instructed to indicate whether the stimulus was stationary or rotating, irrespective of its direction, by pressing one of the two arrow keys. They were informed that the stationary stimulus would be presented on half of the trials and the rotating stimulus on the other half.

#### Procedure and Design

There were eight experimental blocks of trials in each session. An additional first block of practice trials was excluded from analyses. Each experimental block was comprised of 48 trials except Session 3, where each block was comprised of 60 trials, so that we gathered 96 trials for each of the experimental conditions in a session. Responses were assigned values of 0, 1, 2, or 3, based on participants’ key responses (see earlier). The value of the clarity rating served as the dependent variable to quantify the perceived illusory rotations. On trials where the rotating inducer was shown, the direction indicated by the keypress was compared to the actual rotation direction to determine congruency rates.

#### Statistical Analyses

In each experimental session, RRI clarity ratings were collapsed across the clockwise and counterclockwise directions, and the average clarity rating was determined in each stimulus condition. The effects of the independent variables on the averaged ratings were evaluated by two-factor repeated measures ANOVAs that combined the factor *Inducer Type* (rotating vs. stationary) with *Number of Points*, *Diameter*, or *ISI*. Participants’ ability to distinguish between the rapidly rotating inducer and the stationary inducer was analyzed by a signal detection analysis (see Experiment 2). In addition, we examined the effects of *Number of Points, Diameter*, and *ISI* on congruency rates to determine how these variables affected the MBE.

Unexpectedly, in contrast to the results obtained in Experiment 2, in the final session of Experiment 3 when 16 points were rotated at 1500°/s, participants reported a rotation slightly more often when a rotating inducer was shown than when a stationary inducer was shown, indicating they could distinguish between the inducer types at a better than chance level. We therefore performed additional analyses in which the data from the three initial sessions were reanalyzed with an ANOVA that included the between-subjects factor *Inducer Sensitivity* (poor vs. good). These analyses are reported in the section on “Sensitivity to Inducer Motion.” All reported ANOVA *p* values were Greenhouse–Geisser corrected, but for the sake of readability, we report the uncorrected degrees of freedom.

### Results

**Figure 5.**
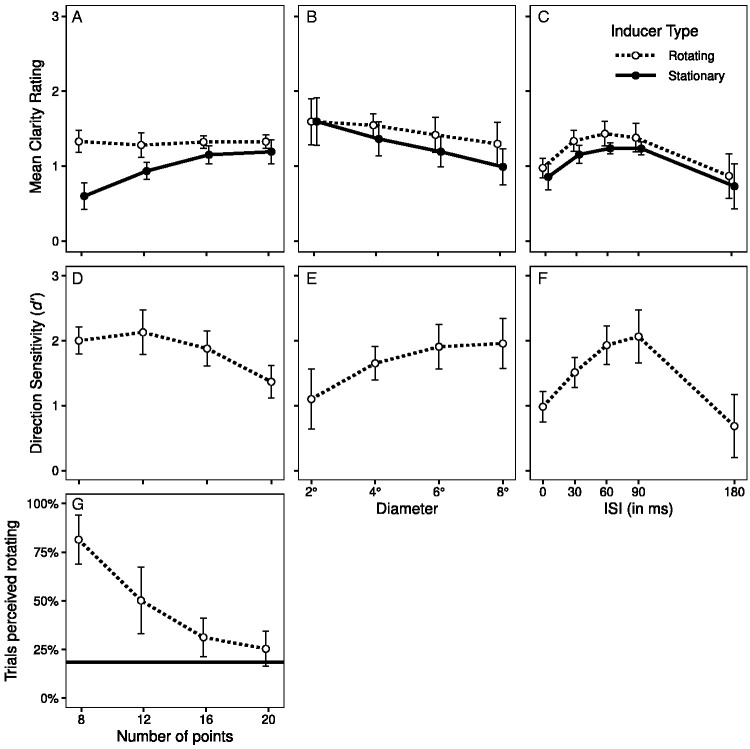
Measures of the Ring Rotation Illusion and the Motion Bridging Effect in Experiment 3. Top row: mean rating of clarity of the illusionary rotation with rapidly rotating and stationary inducers as a function of (A) Number of Points, (B) Diameter, and (C) the inducer-test ring interstimulus interval (ISI). Middle row: sensitivity to the congruence of the rotating inducing ring and perceived test ring rotation directions (the MBE) as a function of (D) Number of Points, (E) Diameter, and (F) the inducer-test ring ISI. Bottom row: (G) mean frequency of perceiving the inducer alone as rotating or stationary as a function of number of points and inducer type. Because Number of Points could only be varied with the rotating inducer, the black line indicates the overall incidence of reports of rotation when the stationary inducer was shown. In A–F, the stationary test ring followed the inducer. In G, the inducer appeared alone. Error bars in A–F show the 95% confidence intervals for the within-subject difference in means, in G for single means.

Results are presented in Figure 5. Across Sessions 1, 2, and 3, clockwise and counterclockwise rotations were, respectively, reported on 45.45% and 24.33% of the trials with the stationary inducer and 48.84% and 32.17% of the MBE trials with the rotating inducer. On the remaining 30.22% of stationary inducer trials and 18.99% of rotating inducer trials, participants reported they could not specify a motion direction. As noted in our presentation of Experiment 1, this could have occurred because they did not perceive any motion or because there was more than a single rotation direction perceived. The difference in the frequency of these reports proved significant on a two-tailed Wilcoxon signed-rank test, *W*(11) = 76, *p* = .003.

#### Number of Points

*Number of Points* had a significant main effect on the clarity of the test ring’s perceived rotation, *F*(3, 33) = 16.82, *p* < .001, η_p_^2^ = .605 (Figure 5A). Clarity increased as the number of points in the rings increased. The main effect of *Inducer Type* was also significant, *F*(1, 11) = 17.51, *p* = .002, η_p_^2^ = .614, indicating clearer motion percepts with the rotating than the stationary inducer, an outcome also observed in Experiment 2. In addition, there was a significant *Inducer Type* × *Number of Points* interaction, *F*(3, 33) = 17.65, *p* < .001, η_p_^2^ = .616. Separate post hoc analyses confirmed what is apparent in Figure 5A: This interaction occurs because the improvement in motion clarity occurs only with the stationary inducer, *F*(3, 33) = 26.50, *p* < .001, η_p_^2^ = .707. Clarity is poorer with the stationary inducer than the rotating inducer when the number of points is low but improves as number of points increases until at the highest *point-number* tested the clarity is almost identical with the two inducer types. *Number of Points* has no effect on clarity when the rotating inducer is shown, *F*(3, 33) = 0.32, *p* = .739, η_p_^2^ = .029.

The effect of *Number of Points* on the MBE congruency effect when the rotating inducer was presented was quantified in terms of *d ′* values (Figure 5D). An ANOVA confirmed that the main effect of *Number of Points* on MBE congruence was significant, *F*(3, 33) = 7.30, *p* = .002, η_p_^2^ = .399, with the MBE *decreasing* as the number of points increased. This finding replicates a previous finding by Stein et al. (2019) but contrasts with both the null effect of *Number of Points* on motion clarity ratings following presentations of the rotating inducer and the positive effect on clarity following presentations of the stationary inducer (Figure 5A).

#### Diameter

Visual inspection of Figure 5B suggests the clarity of the illusory test ring rotation gradually diminished as the diameter of the rings increased, but the main effect of *Diameter* did not reach statistical significance, *F*(3, 33) = 2.99, *p* = .084, η_p_^2^ = .213. However, the main effect of *Inducer Type* was once again significant, *F*(1, 11) = 5.82, *p* = .035, η_p_^2^ = .346, indicating that there was a clearer RRI with the rotating inducer (Figure 5B), and this effect significantly interacted with *Diameter*, *F*(3, 33) = 5.75, *p* = .007, η_p_^2^ = .343. Post hoc analyses revealed this interaction occurred because *Diameter* produced a significant reduction in the motion clarity when the stationary inducer was shown, *F*(3, 33) = 5.03, *p* = .015, η_p_^2^ = .314, but not when the rotating inducer was shown, *F*(3, 33) = 1.31, *p* = .285, η_p_^2^ = .107. Note, that in Experiment 1, where the stimulus duration was 500 ms, a similar but nonsignificant reduction in RRI clarity was found (see Figure 2B).

As shown in Figure 5E, the MBE congruency effect *increased* as a function of *Diameter*. This increase was significant, *F*(3, 33) = 5.46, *p* = .025, η_p_^2^ = .332. This finding contrasts with both the null effect of *Diameter* on motion clarity following the rotating inducer and the *decrease* in clarity following the stationary inducer (Figure 5B) but replicates a previous finding by Stein et al. (2019).

#### ISI

The main effect of *ISI* on the clarity of the test ring motion was significant, *F*(4, 44) = 9.57, *p* = .003, η_p_^2^ = .465. Figure 5C shows a clear inverted U-shaped function that indicates the illusion was maximal with an ISI of 60-90 ms. This function replicates the one found in Experiment 1 when the inducer presentation was brief. As in Experiment 2, the main effect of *Inducer Type* was also significant, *F*(1, 11) = 9.01, *p* = .012, η_p_^2^ = .450, with the rotating inducer producing a clearer motion percept than the stationary inducer. Importantly, the interaction *Inducer Type* × *ISI* did not reach significance, *p* = .77, indicating that the effect of ISI was similar with the two inducer types.

Figure 5F shows MBE congruency was also an inverted U-shaped function of ISI with a maximum when the ISI is 90 ms. ANOVA results confirmed the significance of this effect, *F*(4, 44) = 14.34, *p* < .001. This function not only accords with the present clarity outcomes (Figure 5C) but also with a previous finding by Mattler and Fendrich (2010).

#### Sensitivity to Inducer Motion

Figure 5G shows the frequency participants reported seeing a rotation as a function of *Number of Points* when the rotating or the stationary inducer was shown without the test ring. The main effect of *Number of Points* on *d ′* was significant, *F*(3, 33) = 52.41, *p* < .001, η_p_^2^ = .827, with mean values of *d ′* = 2.22, *d ′* = 1.07, *d ′* = 0.52, and *d ′* = 0.33 for 8, 12, 16, and 20 points, respectively. Post hoc one-tailed *t* tests revealed that mean sensitivity was above chance in all cases (all *p* < .01).

The finding that participants’ sometimes discriminated the stationary from the rotating inducer in the standard 16-point condition, *t*(11) = 4.92, *p* < .001, distinguishes this result from the one obtained in Experiment 2. The source of this discrepancy is not clear to us: It could reflect individual differences in the groups of participants in the two experiments, or differences in the experimental contexts. We note with regard to the latter possibility that in Experiment 3 conditions where the inducer was constructed with less than 16 points improved the ability of the participants to detect rotations. These trials may have trained subjects to attend to subtle motion cues. But whatever the source of this discrepancy, it raises the possibility that an ability to distinguish between the inducer types contributed to the effects of *Inducer Type* in Experiment 3. To assess this possibility, we first examined the correlation between each participant’s sensitivity to the presence of an inducer motion (*d ′*) and the size of the effect of *Inducer Type* on their clarity ratings at each of the tested number of points settings. We reasoned that if the effect of *Inducer Type* on the clarity of the RRI (Figure 5A) was produced by differences in the perception of the inducer (Figure 5G), this would lead to a correlation between the two measures. Across participants, *d ′* values for the discrimination of inducer type and clarity rating differences correlated with *r* = .60, .38, –.14, and –.18 (*p* = .04, .22, .66, and .57) for trials with 8, 12, 16, and 20 points, respectively. Thus, no correlation occurred in the 16-point condition (see scatterplots illustrating these outcomes in supplementary Figure S1).

In addition, we divided our participants into two subgroups based on their ability to distinguish the inducer types in the 16-point condition: a poor performance subgroup (mean *d ′* = 0.205) and a good performance subgroup (mean *d ′* = 0.841). Then, we conducted additional ANOVAs, which included the between-subjects factor *Inducer Sensitivity* (poor vs. good), to see whether this factor influenced reports of motion clarity. No statistically significant main effect of *Inducer Sensitivity* on motion clarity was found in any of the extended ANOVAs (*p* = .11 or greater in all tests), and, importantly, there was no interaction of *Inducer Sensitivity* with the effect of *Inducer Ty*pe (*p* = .12 or greater in all tests). Likewise, all of the interactions of *Inducer Sensitivity* with *Number of Points*, *Diameter*, and *ISI* were nonsignificant (*p* = .14 or greater). Thus, we found no evidence that perceived differences between the stationary and rotating inducers had an effect on ratings of the clarity of the RRI in the initial three sessions.^2^

#### The Relation Between Direction Sensitivity and Motion Clarity

**Figure 6.**
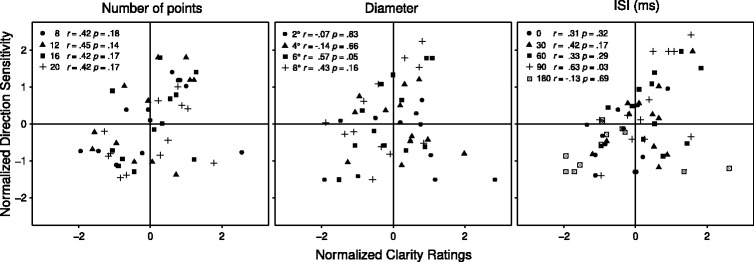
Relation between the congruency effect and motion clarity in the MBE trials of Experiment 3. Individual *d*′ values of the congruency effect and individual clarity ratings are normalized to M = 0 for each experimental condition. Left panel: data of the session in which the number of points was varied between trials. Middle panel: data of the session in which the diameter of the rings was varied blockwise. Right panel: data of the session in which the inducer-test ring interstimulus interval (ISI) was varied between trials. Symbols signify the levels of the independent variable.

To further examine the relationship between sensitivity to motion direction in the MBE trials (Figure 5D to F) and the motion clarity ratings in those trials (Figure 5A to C), we evaluated whether participants’ ability to detect the motion direction was correlated with their clarity ratings. To clarify the graphical presentation of these analyses, we normalized both the direction sensitivity and clarity values to M = 0 and computed the correlation between these measures for each participant in each experimental condition of Experiment 3. The scatterplots in Figure 6 depict these correlations. Values of *r* range from –0.13 to 0.63 with a Fisher *z*-transformed average of *r* = .33. The variability of these correlations and the fact that all but two of them are nonsignificant support the view that the MBE congruency effect and the perceived clarity of test ring rotation result from separable processes. The fact the 11 of the 13 correlations are positive, however, suggests the two processes may interact (see the General Discussion section).

### Summary

Experiment 3 compared the stimulus dependencies of the motion illusions produced by stationary inducer displays and MBE displays. As in Experiments 2, motion clarity ratings are slightly but significantly higher and reports of no or bidirectional motion less frequent with the MBE display than with the stationary inducer display. Unexpectedly, participants had a limited ability to distinguish the stationary inducer from a rotating inducer with 16 points in Experiment 3, although they could not distinguish identical stimuli in Experiment 2. However, our analyses revealed no evidence that this capability was related to the finding that there was greater motion clarity with rotating inducers.

The same distinctive inverted U-shaped motion clarity function is found with the two display types as the ISI between the inducer and test ring presentations gets longer. This is commensurate with the hypothesis that the RRI and MBE motion percepts are at least partially attributable to a common mechanism, as one would expect given the similarity in the appearance of the displays and the illusions they produce. However, the other stimulus dependencies we investigated are not identical for the two display types. A reduction in motion clarity with increasing ring diameter that occurs with the stationary inducer display is strongly attenuated with the MBE display, and an improvement in motion clarity as test ring point-number is increased occurs with the stationary inducer display but not with the MBE display. Furthermore, ring diameter and test ring point-number both modulate the MBE congruency effect in the reverse direction of their modulation of RRI motion clarity. Finally, there was no consistent relationship between participants’ sensitivity to the motion direction of the rotating inducer – the congruency effect – and the clarity of the perceived motion. Together, these outcomes suggest that the MBE congruence effect is mediated by a separate mechanism than the one that generates the RRI motion percept, and the MBE inducer spin does not just bias the direction of illusory rotation but also acts to augment and support that illusion.

## General Discussion

In this study, we introduced the “Ring Rotation Illusion,” which is defined as the illusory rotation of a stationary ring of points when it replaces a stationary outline circle. The stationary inducer display is very similar to the display that generates the MBE – the illusory rotation of a stationary ring of points that replaces a ring of points that rotates so rapidly it appears to be a continuous outline. The MBE is distinguished by the fact that the direction of the initial rotating ring determines the direction of the subsequent illusory rotation. We compared the RRI and MBE and found not only functional similarities but also crucial differences, which suggest that the two illusions cannot be considered equivalent, although a common mechanism may be operating in the generation of the rotation percept in both cases. In the following discussion, we compare the RRI to instances of apparent motion, address the relation between the RRI and the MBE, consider possible mechanisms that could determine the direction of the illusory rotation, and note the limitations of our measure of the illusory motion.

### The RRI and Apparent Motion

The display that generates the RRI bears a resemblance to the displays used to produce another illusion, which has been variously termed Singularbewegung (Wertheimer, 1912), polarized gamma movement (Kanizsa, 1979), the line-motion illusion (e.g., Hikosaka et al., 1993), motion induction (e.g., von Grünau & Faubert, 1994), and illusory line motion (e.g., Kawahara et al., 1996). We will adopt the designation “line motion illusion” with its “LMI” acronym.

In LMI experiments, an initial target shape (typically a dot or small square) is followed by the presentation of an adjacent spatially extended shape (typically a line or elongated rectangle). For convenience, we will refer to the initial shape as the *cue* and to the second shape as the *line.* Observers perceive the line to form by growing rapidly outward from the cue. Multiple instances of the LMI in a spatial array can be seen to occur simultaneously (Faubert & von Grünau, 1995; Kawahara et al., 1996; Tang et al., 2013). Moreover, the order of the cue and line presentations can be reversed, in which case the line is seen to contract into the cue (e.g., Downing & Treisman, 1997). This latter case highlights the RRI’s similarity to the LMI: in both illusions, switching between one or more spatially extended stimuli (the line or lines in the case of the LMI and the outline circle in the case of the RRI) and one or more localized discrete stimuli (the cue or cues in the case of the LMI and test ring points in the case of the RRI) creates an illusory motion.

Contemporary research on the LMI was initiated by Hikosaka et al. (1993) who regarded it as an attention-related phenomenon. However, Downing and Treisman’s (1997) finding that participants continued to perceive a smooth illusory motion when they reversed the order of the line and cue (so that attention is not drawn to an initial focal point) and demonstrations that the LMI can occur at multiple locations simultaneously (Faubert & von Grünau, 1995; Kawahara et al., 1996; Tang et al., 2013) are difficult to reconcile with an attention-based account. In addition, studies that used endogenous (Christie & Klein, 2005) and exogenous spatial cues (Fuller & Carrasco, 2009) to create shifts of attention have failed to find these cues had any effect on the LMI. Low-level processes based on shifts in luminance or contrast profiles have also been proposed to explain the LMI (Hock & Nichols, 2010; Zanker, 1994). However, low-level models of this kind cannot easily account for instances with complex LMI displays where motions are perceived that violate the predictions made by motion energy models (e.g., Tse & Logothetis, 2002; see the review in Han et al., 2016).

Downing and Treisman (1997) offer a high-level alternative to account for the LMI. They relate the LMI to apparent motion and suggest it represents a case of an “impletion” process that fills in the missing intermediary positions of objects undergoing apparent motion. The LMI is regarded as “a progressive transformation of shape induced by the binding of the cue and the line as successive states of one moving object” (p. 777). Downing and Treisman suggest the impletion process reflects a visual inference that interprets a stimulus sequence in terms of the real-world state of affairs likely to produce it. Hsieh et al. (2005) expand on this idea and speak of visual “heuristics.” These high-level accounts treat the LMI as an instance of what Tse et al. have termed *transformational apparent motion* (TAM; Tse et al., 1998; Tse & Logothetis, 2002). This is the motion percept that occurs when a visual figure is suddenly replaced by another spatially overlapping figure and observers see the first figure morphing smoothly into the second.

The RRI might be viewed as a global version of the LMI in which multiple segments of the outline of the circular inducer contract in unison into the circular array of points that form the test ring. A study by Tang et al. (2013) provides support for this view. These investigators studied circular arrays of various synchronous TAM stimuli of the LMI type. They found that multiple TAM stimuli could integrate to yield a coherent global motion percept. The finding that grouped TAM arrays can generate a global motion percept accords with the hypothesis that in the case of the RRI, a local contraction of segments of the inducer into the test ring points generates a global percept of rotation.

This hypothesis, however, raises the new problem of explaining why all the points in the RRI test ring yoke and rotate together in a common direction. The global rotations reported by Tang et al. (2013) were supported by directional cues that the local TAM elements in their displays provided. In the case of the RRI, however, no local directional cues are present. A plausible percept in the case of the RRI would be to see the segments of the inducing ring adjacent to each of the test ring points contracting symmetrically into those points. Downing and Treisman (1997) reported just such a percept with an LMI display: When a line presentation was followed by two cues, one at each of its ends, the line was seen to divide at its center and contract into both the cues. We have not, however, observed a percept of this sort in this or any of our prior investigations, and no participant has reported such a percept.

Another variant of the LMI reported by Downing and Treisman (1997) bears on the yoking issue. When two horizontally aligned cues were followed by two lines, one between the cues and one to the right of the rightward cue, both cues were seen to expand rightward to form the lines (see also Faubert & von Grünau, 1995). This suggests a perceptual yoking of the perceived motion directions. Yoking across discrete instances of apparent motion has also been reported by Ramachandran and Anstis (1983). These investigators presented bistable pairs of points that could be seen to be undergoing either a horizontal or vertical oscillation when repetitively switched between their alternative states. When groups of these point-pairs were presented and switched simultaneously, the same perceived direction of the oscillation was entrained into all the pairs. Ramachandran and Anstis (1983) suggest that their finding provides support for the view that their display is perceptually interpreted as a single object moving through space. This interpretation could also be applied to the RRI display and would provide a plausible account for our finding of a common direction of the rotation of all the test ring points.

The character of the RRI’s dependency on ISI may distinguish it, however, from the LMI. Following brief inducer presentations, the clarity of the RRI motion is not at its maximum when the ISI between the test and inducing ring is zero: It rather increases to a maximum with an ISI of 60–90 ms, and then decreases as the ISI grows longer. We are aware of only one LMI study which inserted an ISI between the presentations of the cue and line. Hikosaka et al. (1993) coupled a cue that was flashed for only 2 ms with cue-to-line stimulus-onset asynchronies (SOAs) ranging from 1 to 4,800 ms so that all but their shortest SOAs were effectively also ISIs. They found the LMI was maximal with SOA of about 100 ms and for four out of five observers remained at this level until the SOA exceeded 400 ms. The difference in the profiles of the RRI and LMI as ISI increases suggests that the two illusions may not have a common basis.^3^ It should be acknowledged, however, that the MBE congruency effect is modulated by ISI in the same way as clarity is, so the pattern we observed could be attributable to a general modulation of the coupling between the inducing and test rings rather than the modulation of a specific motion generating process.

Most important, the character of the motion percept distinguishes the RRI from the LMI. In the many LMI variations that have been reported, it is always the line stimulus that hosts the motion percept, either by growing or contracting. In the RRI, however, it is the points of the test ring that host the motion percept by appearing to rotate. To the best of our knowledge, there is no LMI counterpart to this. The global rotational movement reported by Tang et al. (2013) comes closest, but in their displays, the global percept is also conveyed by visible local line segments.

To sum up, accounting for the RRI remains a challenge. It bears a similarly to the LMI, but the limited data available suggest its temporal dependency on ISI is different from that found with the LMI. Moreover, the RRI differs from all reported instances of the LMI because the motion percept is conveyed by the rotation of the points and not the growth or shrinking of a line. Thus, whether the RRI is in fact a novel form of TAM or a completely new type of illusory motion is not entirely clear. In either case, a high-level approach to explaining the RRI that we think merits serious consideration is the visual “heuristics” model proposed by Hsieh et al. (2005). Some examples of possible “heuristics” mentioned by Hsieh et al. are that objects tend to change shape continuously, rarely appear out of nowhere, and rarely disappear into thin air. In the case of the RRI, the argument would be the visual system generates a percept that fits with a heuristic that holds that the real-world event most likely to account for an outline circle suddenly becoming a ring of points is a rapid rotation suddenly coming to a halt. Explanations based on lower level motion processing mechanisms cannot, however, be ruled out.

### The Relation Between the RRI and the MBE

The observed pattern of results appears compatible with the view that the perceived illusory motion of the test ring in the MBE and RRI displays is at least in part generated by a common mechanism, but in the case of the MBE, there is a second mechanism produced by the inducing ring rotation, which although not consciously visible acts to bias the direction of the illusory motion. We propose this second mechanism interacts with the primary motion generating mechanism in a manner that not only biases the perceived motion direction but also augments the strength of the illusion of rotation.

We see at least two ways this augmentation might occur. One possibility is that the motion direction signal conveyed by the rapidly rotating inducer directly supplements or bolsters the primary process. This could be occurring at a relatively low level in the visual system. An alternative high-level account is also plausible. Note that the direction of the perceived rotation in the case of the RRI is inherently ambiguous. If the spin of the inducing ring with the MBE display helps to resolve this ambiguity, an overall impression of greater motion clarity could result. This account would lead one to expect the greatest clarity when the direction of the test ring and inducing ring motions are congruent, which is in fact what we observed.

If variations in the ring diameter and number of points that form the rings weaken and strengthen the MBE motion biasing process in the reverse direction of their effect on the primary motion generating process, as the congruency data indicates (Figure 5D and E), this could account for the reduced effect of ring diameter and number of points with the MBE display relative to the stationary inducer display. Specifically, the benefit for the RRI produced by the increase in test ring number of points could be countered by an associated loss of the putative motion augmentation generated by the second process, and the weakening of the RRI motion percept with increased diameter could be countered by an increase in motion augmentation by the second process.

We note in this regard that opposing modulations might be mediated by mechanisms operating on different components of the MBE display. To keep the number of points that formed the MBE inducing and test rings the same, an increase in the number of test ring points was always accompanied by a matching increase in the number of inducing ring points. While the improvement in clarity of the illusory motion observed with the stationary inducer display when the number of test ring points was raised can only be attributed to the test ring change, the associated reduction in the strength of the motion biasing mechanism with the MBE display can be attributed instead to the increase in the number of inducing ring points. This could occur because increasing the number of inducing ring points increases the temporal frequency of the inducing ring point presentations as the inducer rotates and/or because it decreases the spacing between the inducing ring points. Both these possibilities are addressed in Stein et al. (2019).

### The Spatial Relationship Between the Inducing and Test Rings

While the present report does not investigate the extent to which the RRI depends upon the precision of the spatial overlap of the inducing and test rings, some data regarding the effect of spatial overlap in the case of the MBE are provided in the 2010 report of the MBE by Mattler and Fendrich. In this study, Test Procedures 6 to 9 in the section on phenomenological testing investigated the effects of making the test ring diameter 1° or 3° of visual angle larger than the inducing ring and the effect of displacing the test ring position 1° or 3° upward from the inducing ring. In these tests, a rotating inducer with a velocity of 1500°/s and 90 ms duration was presented, followed by an ISI of 60 ms, followed by a 500 ms test ring. With these parameters, it had been previously found that when the positions of the test and inducing rings were superimposed, an inducing ring motion was almost always seen and the illusory test ring rotation matched the direction of the inducing ring on 77% of the trials. When the test ring diameter was 1° larger than the inducing ring, motion was seen on the 96% of the trials and a congruent motion on 55% of the trials. When the test ring diameter was 3° larger than the inducing ring, motion was seen on 68% of the trials and a congruent motion on 35% of the trials. When the test ring was displaced 1° upward, motion was seen on 97% of the trials and a congruent motion on 58% of the trials. When the test ring was displaced 3° upward, motion was seen on 92% of the trials and a congruent motion on 49% of the trials.

Thus, a 1° position mismatch between the inducing and test ring positions substantially degraded the MBE congruency effect and a 3° mismatch totally abolished it. However, the incidence of the motion percepts was largely unaffected by the 1° position mismatch and reduced but not eliminated by a 3° mismatch. These outcomes provide additional support for the hypothesis that the illusory test ring rotation observed with both the RRI and MBE and the direction biasing effect that characterizes the MBE are produced by separable processes, with the processes that produce the test ring rotation being considerably more tolerant of spatial position mismatches than the processes that produce the direction biasing.

If this is the case, a small spatial misalignment between the inducing and test rings should eliminate the improvement in motion clarity found with rotating relative to stationary inducers, but the clarity of the RRI should be largely unaffected by the misalignment. Further research could readily evaluate this prediction. In addition, we note that we have made experimental observations that indicate the congruency effect observed with the MBE is exceedingly sensitive to the spatial alignment of the end position of the inducing ring points with the positions of the subsequent test ring points when the test ring replaces the rotating inducing ring – to the extent that certain misalignment conditions can actually reverse the biasing direction (Stein, 2020).

### The Direction of Perceived Rotation

In the case of the MBE, the perceived direction of rotation usually matches the direction of the rotating inducer. However, in all our experiments with both RRI and MBE, participants reported clockwise rotations more frequently than counterclockwise rotations. While there were substantial individual differences in the strength of this clockwise bias, with a given participant, the strength of the bias tended to persist across the experimental sessions. Overall this bias was not conspicuously different in the 29 right and 7 left-handed participants, who respectively reported clockwise rotations on 64.9% and 72.2% of the trials in which the motion direction was specified. A similar clockwise rotational bias has been found in the ‘silhouette illusion’ created by Nobuyuki Kayahara (Troje and McAdam, 2010; see Wexler, Duyck, & Mamassian, 2015, and Karim, Proulx, & Likova, 2016, for examples of directional biases in other perceptual and motor tasks). We do not know the source of this clockwise bias, but top-down effects related to attention and expectations seem possible. There may, for instance, be a perceptual bias produced by our familiarity with clockwise rotations due to our experiences with analog clocks. Commensurate with this speculation, Morikawa and McBeath (1992) have shown that reading direction can exert a robust bias on the direction of an ambiguous lateral apparent motion. These considerations point to the general need for further research on the character of the signals that determine the direction of ambiguous motions.^4^

### Measures of the Strength of the Illusory Motion

Measuring the subjectively perceived properties of visual illusions is challenging. Here, we employed a clarity rating that has been used in previous research on apparent motion (e.g., Kahneman, 1967) and the LMI (e.g., Christie & Klein, 2005; Downing & Treisman, 1997; Hubbard & Ruppel, 2011, 2018). This measure has been shown to be statistically robust (Norman, 2010). The systematic variations in the clarity ratings we found in Experiment 1 were replicated in Experiment 3, which argues for the veracity of the rating patterns we observed.

Nevertheless, clarity is an inherently subjective and therefore somewhat fuzzy metric and can be based on idiosyncratic criteria. A replication of the present findings with objectively quantifiable psychophysical measures of the illusion would therefore be desirable. We are currently working to develop such alternative approaches. Cancellation techniques in which real motions are used to null illusory motions offer one possibility (e.g., Blake & Hiris, 1993; Han et al., 2016; Steinman et al., 1995; Wright & Johnston, 1985). Han et al. used this approach to quantify the motion seen in the LMI (see also Fuller & Carrasco, 2009; Ha & Hamm, 2019). Measures that take advantage of the fact that moving stimuli can shift the perceived position of stationary stimuli offer another possibility (e.g., Durant & Johnston, 2004; Faubert & von Grünau, 1995; Scarfe & Johnston, 2010; Whitney, 2006). The actual displacement that is necessary to compensate the motion-induced displacement of the two stimuli can be used as a measure to quantify perceived motion. Whitney (2006) has used this approach to study illusory line motions. Comparison techniques might also be employed. For example, the illusory rotation might be compared to an actual rotation, or the end time of the illusory rotation might be compared to the offset time of an external reference. Displays that use this last approach are being evaluated in our lab.

## Conclusion

We report evidence for a new illusion, the “Ring Rotation Illusion,” which occurs when a circular outline is followed by a stationary ring of points. This can produce a brief but compelling illusory rotation of that ring of points in one direction. We quantified the strength of this illusion by subjective ratings of the clarity of the rotation. Mean clarity was modulated by the number of points in the stationary ring, the diameter of the rings, the ISI between rings, and the duration of the rings. We compare the RRI with the MBE, a similar illusion in which the inducer is a ring of points that rotates so rapidly it appears to be a continuous outline. We find the similarity of the two illusions is commensurate with their sharing a common generative mechanism. However, a crucial characteristic of the MBE, the congruence between the direction of the inducer’s spin and the apparent test ring rotation, is necessarily dependent on the inducer spin and therefore has no RRI counterpart. This rotation-dependent mechanism appears to influence the perceived clarity of the MBE motion percept as well as its direction, augmenting that clarity and potentially countering the functional dependencies found with the RRI. We suggest that the RRI is a variant of apparent motion, which may be the result of a visual heuristic that interprets the stimulus sequence in a manner that accords with a conjectured sequence of events in the real world.

## Supplemental Material

sj-pdf-1-ipe-10.1177_20416695211020019 - Supplemental material for The Ring Rotation Illusion: Properties and Links of a Novel Illusion of Motion Click here for additional data file. Supplemental material, sj-pdf-1-ipe-10.1177_20416695211020019 for The Ring Rotation Illusion: Properties and Links of a Novel Illusion of Motion by Uwe Mattler, Maximilian Stein and Robert Fendrich in i-Perception

## Notes

1 To additionally confirm that individual differences in participants’ sensitivity to the inducer type did not modulate the effect of inducer type on their clarity and distance judgments, we correlated participants’ sensitivity levels with the effect of inducer type of their clarity and distance ratings in the 1500°/s condition. These correlations were not significant (clarity: *r* = –.25, *p* = .43; distance: *r* = –.21, *p* = .51).

2 A reviewer raised the issue whether eye movements could play a role in the inducer discrimination task. This is in principle possible. Saccades (including microsaccades), for instance, could briefly shift the retinal path along which moving inducer stimuli advance, producing systematic local distortions with a rotating inducer not present with a stationary inducer. The orientation of the retinal traces painted during saccades could also reveal a moving inducer’s rotation direction. However, these effects can only occur if participants move their eyes during the brief presentations of the inducer (91 ms and 121 ms in Experiment 2 and 3, respectively). Moreover, we deem it very unlikely that saccades are contributing in a major way to the MBE as the information provided by saccades would be available irrespective of whether the test ring is presented, but judgments of the rotation direction are only above chance when the test ring follows the inducer. In the case of the RRI, the inducer is stationary, so there is no clear reason why eye movements would have any effect.

3 We do not know of any study that examined the effect of ISI on instances of TAM other than the LMI, but note that Tse and Logothetis (2002) wrote: “TAM, in contrast, does not require a blank ISI between images to see the results of form-based matching and may actually be diminished by such an ISI” (p. 258). If this is true, it bolsters evidence the RRI and TAM differ.

4 We note that Wexler et al. (2013) have proposed that in the case of another rotation illusion they call the high-phi phenomenon, the direction of an illusory rotation is determined by a “motion seed” that can link to broadband motion energy. It is possible a similar hypothesis could be applied to the RRI and MBE.
