# Supplementary material for: The Ring Rotation Illusion: Properties and Links of a Novel Illusion of Motion
Source: Iperception. 2021 Jun 7;12(3):20416695211020019. doi: 10.1177/20416695211020019 (PMC8191087; doi:10.1177/20416695211020019)
Supplement: sj-pdf-1-ipe-10.1177_20416695211020019 - Supplemental material for The Ring Rotation Illusion: Properties and Links of a Novel Illusion of Motion [file sj-pdf-1-ipe-10.1177_20416695211020019.pdf]

Supplementary Material

to

The Ring Rotation Illusion: Properties and links of a novel illusion of motion

by Uwe Mattler, Maximilian Stein, & Robert Fendrich

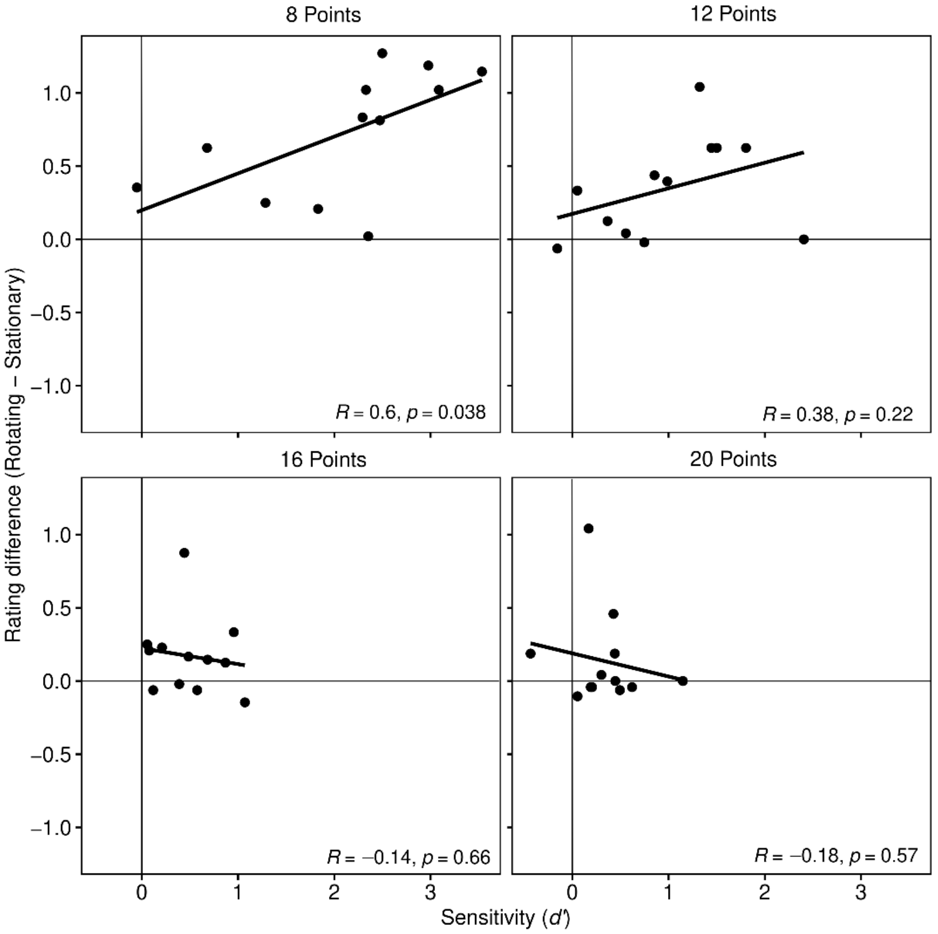

Figure S1. Scatterplots of the relationship between participants' sensitivity (d') when distinguishing between the rotating and stationary inducers and the difference between their clarity ratings with the rotating and stationary inducers in the four number of point conditions of Experiment 3.
